# The Role of Non-Coding RNAs in the Human Placenta

**DOI:** 10.3390/cells11091588

**Published:** 2022-05-09

**Authors:** Milena Žarković, Franziska Hufsky, Udo R. Markert, Manja Marz

**Affiliations:** 1 RNA Bioinformatics and High-Throughput Analysis, Friedrich Schiller University Jena, Leutragraben 1, 07743 Jena, Germany; milena.zarkovic@uni-jena.de (M.Ž.); franziska.hufsky@uni-jena.de (F.H.); 2 European Virus Bioinformatics Center, Leutragraben 1, 07743 Jena, Germany; 3 Placenta Lab, Department of Obstetrics, University Hospital Jena, Am Klinikum 1, 07747 Jena, Germany; udo.markert@med.uni-jena.de; 4 FLI Leibniz Institute for Age Research, Beutenbergstraße 11, 07745 Jena, Germany; 5 Aging Research Center (ARC), 07745 Jena, Germany

**Keywords:** non-coding RNAs, circRNA, microRNA, pregnancy, placenta, extracellular vesicles

## Abstract

Non-coding RNAs (ncRNAs) play a central and regulatory role in almost all cells, organs, and species, which has been broadly recognized since the human ENCODE project and several other genome projects. Nevertheless, a small fraction of ncRNAs have been identified, and in the placenta they have been investigated very marginally. To date, most examples of ncRNAs which have been identified to be specific for fetal tissues, including placenta, are members of the group of microRNAs (miRNAs). Due to their quantity, it can be expected that the fairly larger group of other ncRNAs exerts far stronger effects than miRNAs. The syncytiotrophoblast of fetal origin forms the interface between fetus and mother, and releases permanently extracellular vesicles (EVs) into the maternal circulation which contain fetal proteins and RNA, including ncRNA, for communication with neighboring and distant maternal cells. Disorders of ncRNA in placental tissue, especially in trophoblast cells, and in EVs seem to be involved in pregnancy disorders, potentially as a cause or consequence. This review summarizes the current knowledge on placental ncRNA, their transport in EVs, and their involvement and pregnancy pathologies, as well as their potential for novel diagnostic tools.

## 1. Introduction

The placenta forms the connection and the barrier between the mother and her fetus. It is one of the organs with the highest variability between different species. It differs in shape, size, number and layers between fetal and maternal blood. Moreover, the hormonal regulation of each trimester of pregnancy and the respective sources of hormones are almost unique in each species. Consequently, these differences include the uniqueness of pathologies in different species as reviewed for mice by Schmidt et al. [1]. Therefore, in this review we focus on the human placenta. It may be assumed that the underlying regulation of placentation occurs on a molecular level, which includes specific proteins and their respective specific gene expression control. A great part of this control acts post-transcriptionally through interactions on the RNA level, mainly via non-coding RNA (ncRNA)-induced RNA degradation and inhibition of translation. The cellular interface between mother and fetus is built by different subtypes of trophoblast cells, which are of fetal origin. The functions of the different trophoblast subtypes are manifold and include two-directional transport of water and a broad spectrum of molecules, hormone production, anchoring of the fetus in the uterus, immunoregulation, angiogenesis and others. Trophoblast cells, and mainly the syncytiotrophoblast, are able to produce different types of extracellular vesicles and release them into the maternal circulation. Trophoblast-derived extracellular vesicles contain cytoplasm components from their cells of origin, including proteins and RNA, and transport them to distant targets [2]. This complex system of factors and their balance seems to be susceptible to disorders that may lead to pregnancy pathologies, but it can be expected that yet unknown or uninvestigated factors are also involved. These may include ncRNAs in trophoblast cells and trophoblast-derived extracellular vesicles which seem to be indispensable for the molecular regulation of normal pregnancies, while their dysregulation may lead to the development of pregnancy-related diseases [3].

**Figure 1:**
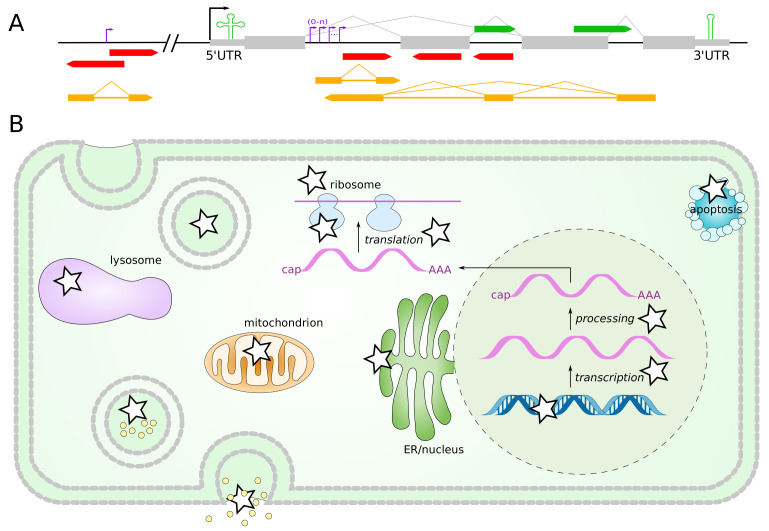
(**A**) Genomic locations of non-coding RNAs. NcRNAs can be located in 5${}^{\prime }$/3${}^{\prime }$ UTRs and introns of proteins and are therewith transcribed with the protein (green). Additionally, ncRNAs exist sense and antisense in introns and exons of proteins and independent of proteins with own transcription start sites and promoter regions (red). Long non-coding RNAs usually span many kilobases and contain their own introns, located sense, antisense or independent of protein coding regions (yellow). (**B**) The various types of ncRNAs function in all basic cellular processes: transcription, processing, translation. They are involved in chromosome structure, DNA replication, gene regulation, genome defense and protein transport as indicated by the stars. Please note miRNA and lncRNAs are located in exosomes and microvesicles.

From the pilot project of ENCODE, we know that less than 3% of the human genome codes for proteins [4,5]. The remaining 97% are divided into 45% repetitive elements (SINEs, LINEs, transposons), 26% introns and other unique non-coding DNA [6]. The question of their meaning has been raised and is only being answered slowly. By now, we know that at least 80% of the human genome has a function [5]. Some ncRNAs are known to be located intergenically, within introns, in 5${}^{\prime }$/3${}^{\prime }$ UTRs, antisense to protein-coding genes or just adjacent to them [7] (Figure 1). Most studies on placental ncRNAs focus on microRNA and long ncRNAs, while studies on ncRNAs of a length in between are comparatively scarce.

miRNAs are 19–25 nt single-stranded RNAs, belonging to the class of small ncRNAs, which mediate the post-transcriptional gene silencing of target RNA transcripts. This gene regulation takes place by binding to mRNA and degrading the transcript or blocking the translation. It is estimated that miRNAs govern the translation of 30–90% of protein-coding genes [8,9]. miRNAs can also be imported back into the nucleus to alter DNA methylation at promoters. Deregulated miRNAs have been associated with all kinds of human diseases.

LncRNAs can act as sponges to inhibit miRNA function by isolating them from their target mRNAs. LncRNAs can further interact with proteins in the cytoplasm and also enter the nucleus to interact with chromatin and regulate gene expression.

The third group of ncRNAs, circular RNAs, are generated from exon regions of protein-coding genes by 5${}^{\prime }$-3${}^{\prime }$ splicing and are proposed to function as sponges blocking miRNA activity. CircRNAs are relatively unexplored in the placenta but are likely to be of importance [10].

During the last decade, microRNAs (miRNAs, 22 nt), as regulators of various cell processes, received major attention. However, according to Rfam 14.6 (July 2021) 4070 RNA families are discovered. About one quarter (1067) are known to exist in humans, covering 16,421 genomic regions (http://rfam.sanger.ac.uk (accessed on 17 January 2022)) [11]. Additionally, the existence of long non-coding RNAs (lncRNAs, >200 nt) containing introns themselves is estimated in humans by GENCODE 7 to 9640 loci [12]. Our knowledge about lncRNAs is limited and only a few general computer programs for their identification are developed, see the section below.

Transcriptome analysis is increasingly used to investigate placental development. Thus, not much is known about ncRNA expression in the human placenta, their functions in placental development, and their role in healthy pregnancies, as well as pathologies ranging from preeclampsia and gestational diabetes mellitus to preterm labor [10]. The number of reports focusing on miRNAs is rapidly increasing (see Morales Prieto and Markert [13], Morales-Prieto et al. [14] for a review) and also placental lncRNAs are brought into focus [15,16,17]. Additionally, the number of studies on placental ncRNAs of a length between miRNAs and lncRNAs has increased during recent years. Here, we focus on a summary of ncRNAs with a known function.

Initially, the principal function of ncRNA was expected to be intracellular regulation. More recently, many studies have demonstrated that ncRNA can be transported via extracellular vesicles which can be incorporated by distant cells of different types where they exert regulative effects [18]. These regulatory functions may be involved in physiological as well as pathological processes [19,20,21].

In this review, we summarize relevant current knowledge on ncRNA and their potential role in the human placenta in healthy and pathological pregnancies.

## 2. Prediction and Identification of ncRNAs

In silico identification of ncRNAs is still a huge challenge. The function of ncRNAs is mainly characterized by their secondary structure rather than by their sequence. Gene identification tools based on sequence homology fail for ncRNAs. Therefore, almost all ncRNA-related computational tools rely on compensatory mutation analysis or covariance models, retaining the secondary structure. The vast majority of ncRNAs are not assigned to a function yet. Whereas many ncRNA classes can be nowadays solidly predicted by secondary structure homology, a de novo detection of ncRNAs in silico is still a bioinformatical challenge. Only a few tools have been developed (Table 1), which, however, are usually restricted by disadvantages, such as high false-positive rates. Therefore, due to their diverse and fast-evolving sequence they are identified most efficiently by a combined ex or in vivo/in silico approach, including sequencing the transcriptome and the usage of established bioinformatical tools with tissue/organ/organism-specific features, such as considering specific promoters, terminators, or transcription signatures.

### 2.1. Identification of miRNAs as Marker Genes

Finding reliable miRNAs as biomarkers and their corresponding targets is a non-trivial task. Potential targets are scored differently by predictive computational tools and miRNA expression and miRNA:target interactions can be tissue- or context-specific but are mostly neglected in databases. Therefore, understanding the limitations of target identification is very relevant.

**Figure 2:**
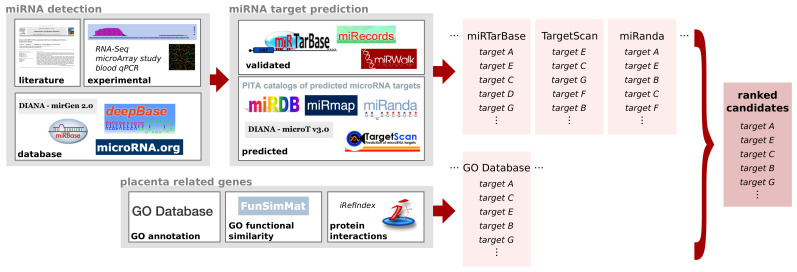
Computational approach to detect microRNAs as potential biomarkers. miRNAs of interest can be detected experimentally and found in the literature or databases. Alternatively miRNAs can be predicted in silico and verified by transcription expression profiles. miRNA target prediction can be performed with various tools, each ranking the possible target by a score, p-value, and other criteria. Placenta-related protein genes can be obtained from databases for annotation, function or protein–protein interaction or other sources, such as own experimental data. Combining all information results in a comprehensive candidate list for miRNA and possible target genes.

The identification of miRNAs and corresponding target genes can be divided into three steps: First, miRNAs of interest are selected based on information from the literature, experimental studies (e.g., high-throughput) or from databases (Figure 2 left). Second, target genes are predicted using various in silico tools for each miRNA (Figure 2 center). Third, a comparison of these possible target genes with genes known to be involved in the disease of interest (Figure 2 bottom panel) completes the computational prediction of miRNAs and their targets. While in silico predictions are fast, inexpensive, and provide a wealth of possible gene connections, predictions can be false-positive. To reduce this risk, as many prediction tools as possible are often combined (Figure 2 right).

In addition, the initial identification of possible marker miRNAs may be biased by a number of factors, e.g., the age of the pregnant women, that may influence miRNA expression levels.

The miRBase database is a searchable database of published miRNA sequences and annotation [35,36]. The Human microRNA Disease Database (HMDD v.3.2, March 2019) is a database that curated experiment-supported evidence for human miRNA and disease associations, and currently contains 1206 miRNA genes in 893 diseases [37,38] (www.cuilab.cn/hmdd/ (accessed on 17 January 2022)), including 156 entries for preeclampsia-related miRNAs.

### 2.2. Non-Coding RNA Target Prediction

The prediction of the ncRNA function is a widely unsolved problem. Non-coding RNAs may interact with other RNA molecules, DNA, or with proteins [39]. The latter one in particular is computationally not explored yet. Interactions with DNA are commonly predicted as interactions with RNAs, just with the slightly different exclusion of GT/UG basepairs. If a specific region of the ncRNA is known to be functional, a vague prediction of its target is possible. However, if this region is smaller than 20 nt (such as for miRNAs), the prediction in general usually comes with a lot of false-positive target candidates. Target prediction of miRNAs, however, has been investigated intensively in the last decade. The prediction of targets in an evolutionary context based on multi-sequence alignments has been described as most reliable [40]. Combined results of predicted and experimentally verified targets can be accessed by various databases (e.g., mirbase). However, a general approach for ncRNA target prediction still needs to be developed.

## 3. MicroRNAs and MicroRNA Clusters in Placenta

Although first described in 1993 [41], only during the last decade have miRNAs emerged as primary epigenetic regulators which have an important role in placental development and function [42]. miRNAs in pregnancy and their complications have been reviewed by Cai et al. [43], Hayder et al. [44], Xu et al. [45]. The role of miRNAs in preimplantation embryo development and the actions of endometrial miRNAs on implantation have been reviewed by Liu et al. [46].

Placental miRNAs have been analyzed in full tissue and in isolated trophoblast cells. As the placenta contains numerous cell types, the results are only partly congruent [47,48,49,50]. In our own studies, we have detected 762 miRNAs in trophoblast cells isolated from the first and third trimester placenta, whereof 382 miRNAs are expressed at relevant levels (Ct <35). When comparing first and third-trimester, 31 miRNAs were up-regulated and 14 miRNAs were down-regulated more than 100-fold [50]. Several miRNAs that are highly expressed in total placenta tissue homogenisate are also highly expressed in isolated trophoblast cells, e.g., miR-21, miR-24, miR-30b, miR-30c, miR-191 and miR-199a [48]. Other placental miRNAs may derive from fibroblasts, immune cells, or endothelial cells. Generally placenta-tissue specific miRNAs can be identified.

### 3.1. miRNA Clusters C14MC, C19MC and miR-371-3 in Trophoblast Cells

A large number of miRNAs is expressed in clusters which almost exclusively appear in trophoblast cells. The two most prominent ones are the largest human miRNA clusters chromosome 14 miRNA cluster (C14MC) and chromosome 19 miRNA cluster (C19MC), supplemented by the miR-371-3 cluster. All of them underly a specific expression kinetic during the course of pregnancy [50]. The functions of each individual miRNA of these clusters are controversially discussed. It may be expected that the coordinated interaction of all members of these clusters will be more powerful than individual members, but analyses of whole cluster functions are still scarce. For more detailed and summarized information refer to [51,52,53,54].

C14MC is located at the DLK1-DIO3 genomic region on chromosome 14 and contains at least 54 different miRNAs [55]. Although a few species divergences have been observed, this cluster is widely conserved among placental mammals. Nonetheless, for several C14MC members no orthologs have been detected in mice (hsa-miR-300, -329-2, -432, -487a, -541, -654, -655, -656, -889, -1185-1, and -1185-2, 376a-2) [56] and vice versa (murine miR-679, -666 and -667) [57]. C14MC members are strongly expressed in the primary first-trimester trophoblast cells and in the immortalized first-trimester extravillous trophoblast-derived cell line HTR-8/SVneo which is a frequently used but also controversially discussed model [50], which has a comparatively stable karyotype, but numerous dissimilarities with primary trophoblast cells [58,59]. The expression of C14MC members decreases towards the third- trimester and they are almost absent in JEG-3 choriocarcinoma cells and their derivates which are still more common models [50,59]. miRNAs of C14CM region are involved mostly in the pathogenesis of cancer [55].

Both, C19MC and the miR-371-3 cluster, are encoded on chromosome 19. They are predominantly expressed in the placenta but also in stem cells [14]. C19MC and C14MC are imprinted in the placenta [60,61]. C19MC consists of at least 46 miRNA genes which are mostly, but not completely conserved among primates but not in other species [14,56]. Members of this cluster are strongly expressed in human placenta tissue where they derive mainly from third-trimester trophoblast cells (less from first-trimester trophoblast) and in JEG-3 cells [47,48,49]. They may also be expressed by other placental cells such as mesenchymal stem cells [62]. C19MC miRNAs are involved in several functions: (1) they regulate implantation through inhibition of epithelial-to-mesenchymal transition [63]; (2) villous stroma and trophoblast cells release EVs containing C19MC and other miRNAs into the maternal circulation [48,49]; (3) their circulation very early in pregnancy suggests a role in the establishment of the maternal–fetal interface [64]; (4) upregulation of C19MC miRNAs is a characteristic phenomenon of preterm birth [65]; (5) C19MC miRNAs are described to be involved in antiviral protection [66]; (6) several C19MC miRNAs may trigger cancer development such as hsa-miR-520c-3p in breast cancer or hsa-miR-519d in hepatocellular carcinoma [67].

The miR-371-3 cluster (including miR-371, -372, and -373) is expressed in primary trophoblast cells, JEG-3 cells, and their hybrids, but not in immortalized HTR-8/SVneo Mice have homologous cluster miR-290-296 to the human miR-371-3 cluster [68]. miRNAs of miR371-3 cluster region act as oncogenes or tumor suppressor when up or down-regulated [69].

### 3.2. Placental MicroRNAs in Pregnancy Pathologies

Placental miRNA may be associated with pregnancy pathologies being involved in their pathomechanism or expressed consequently. For almost all pregnancy diseases, specific miRNA patterns have been described, but sometimes controversially (summarized in [3,70]). miRNAs regulate the proliferation [18,71,72,73], migration [72,74,75,76,77], invasion [18,71,73,74,75,76,77,78,79,80,81,82,83,84] and apoptosis [78,85,86,87] of trophoblast cells and are involved in the development of placental vasculature and spiral artery remodeling [88,89,90].

#### 3.2.1. miRNA and Preeclampsia

In particular, for preeclampsia (PE), the role of miRNAs has emerged over the last five years. Several very comprehensive reviews have been produced [91,92,93,94,95]. However, it is still unclear how miRNAs affect the development and outcome of the disease [96]. In Table 2, we name just a few of the most important miRNAs. miRNAs have been described to be potential biomarkers for onset prediction and development of preeclampsia [96,97].

#### 3.2.2. Gestational Diabetes Mellitus

Poirier et al. [118] reviewed the role of miRNAs in the development of gestational diabetes mellitus (GDM), Iljas et al. [119] with regard to intracellular and extracellular miRNAs and [120] with regard to the role of miR-143 for mitochondrial function and glucose metabolism. Interestingly, the up-regulation of miR-98 in the placental tissue in human GDM is linked to the global DNA methylation via targeting methyl CpG binding protein 2 (MECP2) [121]. Furthermore, placental miRNAs have been shown to potentially contribute to the pathogenesis of GDM through altering trophoblast migration [122], invasion [123,124], and apoptosis [125]. Some miRNAs, such as miR-96-5p and miR-132, can be used as diagnostic biomarkers for GDM [126,127].

#### 3.2.3. Miscarriage

Four human miRNAs were found to be upregulated in tissue samples from early pregnancy loss and all the affected target genes are involved in its pathogenesis [42]. Up-regulation of miR-365 may contribute to recurrent miscarriage by decreasing Serum/glucocorticoid-regulated kinase 1 (SGK1) expression [128]. miRNAs are involved in the pathogenesis of pregnancy loss by the inhibition of trophoblast proliferation [129], migration and invasion [130], as well as by promotion of trophoblast [131,132] and decidual [133] apoptosis, and promotion of immune intolerance [134,135]. The down-regulation of miR-324-3p and KISS1/kisspeptins can serve as biomarker for ectopic pregnancy at early gestational ages [136].

#### 3.2.4. Other Pathologies

In trisomy 21 placentas, seven miRNAs have been verified as upregulated. Three of these miRNAs are located on chromosome 21 [137]. Further, there is evidence that birthweight is regulated by placenta-derived miRNAs [138]. Placental miR-21 and miR-143 are important in the development of macrosomia (fetal overgrowth, birth weight ≥ 4000 g) [139]. Kennedy et al. [140] showed that placental miRNAs can regulate adipokines and affect the birthweight. miR-16-5p, miR-103-3p, and miR-27b-3p can be detected in blood and serve as an early biomarker for fetal growth restriction [141]. Moreover, there is a specific placental miRNA profile in maternal obesity. Some of the affected miRNAs may serve as predictors of lower birth weight and increased postnatal weight gain [142]. The expression of vascular cell adhesion molecule 1 (VCAM-1), the target of miR-590-3p, is reduced in intrahepatic cholestasis in pregnancy [143]. In placenta accreta, miR-29a/b/c and miR-125a inhibit apoptosis of intermediate trophoblast cells at the implantation site by targeting myeloid cell leukemia-1 (MCL1) [144,145]. Two miRNAs (hsa-miR-490-3p and hsa-miR-133a-3p) investigated by Yang et al. [146] showed positive correlation to operation-related blood volume loss. Chen et al. [147] discovered four miRNAs (miR-139-3p, miR-196a-5p, miR-518a-3p, and miR-671-3p) that, together with clinical parameters, can be used for non-invasive prenatal screening of placenta accreta spectrum disorders. In obstetric antiphospholipid syndrome, antiphospholipid antibody-induced up-regulation of trophoblast miR-146a-3p is mediated by Toll-like receptor 4, and miR-146a-3p in turn drives the cells to secrete interleukin-8 by activating the RNA sensor, Toll-like receptor 8 [148].

## 4. Long Non-Coding RNAs in Placenta

The involvement of lncRNAs in the development and function of trophoblast cells and the human placenta has been reviewed by McAninch et al. [149]. Later, a study characterized the lncRNA expression profile in human term placenta and detected the expression of 4463 isoforms from 2899 annotated lncRNA loci, plus 990 putative lncRNA transcripts from 607 intergenic regions [150]. Interestingly, the antisense promoter region of L1PA2, a LINE-1 subfamily, appears to act as a promoter for lncRNAs with placenta-specific expression [151].

The Igf2/H19 gene cluster appears to be the most intensively investigated (reviewed by Nordin et al. [152]). H19 large intergenic ncRNA is not only the most prominent lncRNA in the placenta, but also one of the most highly abundant and conserved transcripts in mammalian development, being expressed in both embryonic and extra-embryonic cell lineages. Its expression is conserved in mice and humans [153]. Maternally expressed H19 is located approximately 130kb downstream of Insulin Like Growth Factor 2 (IGF2) gene, and encodes for an ncRNA which downregulates cellular proliferation [154,155]. H19 is a precursor for miR-675, which targets IGF1R, and thus stalls placental growth during late gestation [156] and also has role in fetal growth restriction [157]. Thus, a certain methylation pattern of H19 exon 1 is closely related to PE and trophoblast abnormalities [158]. H19 is up-regulated in PE, reduces cell viability and promotes autophagy and invasion in trophoblast cells, along with activation of the PI3K/AKT/mTOR pathways [159]. In murine placentas obtained after in vitro fertilization and embryo culture, levels of H19 and IGF2 mRNA are altered, but embryos are phenotypically normal, potentially due to a compensatory process capable of correcting placenta dysfunction [160]. Furthermore, after in vitro fertilization in humans, placental H19 and IGF2 expression is disturbed which may be associated with a loss of imprinting on the paternal allele. H19 and IGF2 expression seem to be negatively correlated [161]. In mice, the expression of IGF2 and H19 was found to be decreased in alcohol-exposed placentas, while, conversely, the expression of H19 was significantly increased in alcohol-exposed embryos [162]. Further, lower expression levels of H19 [157] and differential DNA methylation defects of H19/IGF2 are associated with congenital growth disorders [163]. H19 alters trophoblast cell migration and invasion by regulating TβR3 in placentas with fetal growth restriction [164]. Molar tissues show significant differences in allelic distribution of IGF2 and H19 from normal placenta tissues [165]. The expression of IGF2 is significantly higher in gestational diabetes mellitus, while the expression of H19 is significantly lower in GDM [166].

### Long Non-CODING RNAs in Pathologies

The potential role of lncRNAs in the pathogenesis of PE has been discussed in [167,168,169]. Li et al. [170] identified 78 lncRNAs differentially expressed in GDM and Wu et al. [171] 329 lncRNAs in placenta accreta spectrum disorders. Several lncRNAs are differentially expressed in the placenta of macrosomic fetuses, and may contribute to the pathogenesis [172]. However, specific lncRNAs have been examined in detail (see Table 3).

## 5. Circular RNAs in Placenta

CircRNAs exert their effects by acting as miRNA sponges. CircRNAs in the context of reproduction have been reviewed by Quan and Li [202]. Maass et al. [203] presented a map of human circular RNAs in clinically relevant tissues, including the placenta. A total of 227 circRNAs were found to be significantly up-regulated and 255 circRNAs significantly down-regulated in gestational diabetes mellitus (GDM) and play potential roles in its pathogenesis [204]. Further, circRNAs potentially involved in GDM have been described [204,205]. CircRNAs are also found differentially expressed in preeclampsia [206,207,208,209,210]. Qian et al. [210] described 143 up-regulated and 158 down-regulated circRNAs in PE placental tissues, many of them possessing miR-17 binding sites. The pathomechanism of circRNAs in PE includes sponging miRNAs [211], regulation of trophoblast cells [212], proliferation [213,214], migration [215,216], invasion [217,218,219] epithelial-mesenchymal transition [220] and angiogenesis regulation of vascular endothelial cells [221]. Some circRNA can serve as potential therapeutic targets [222] and biomarkers for PE [223,224] as they can be detected in the blood of pregnant women (see below). Dysregulation of trophoblastic circRNAs is further related with fetal growth restriction [225,226,227], fetal macrosomia [228], and recurrent spontaneous abortion [229].

## 6. Circulating Non-Coding RNAs in Maternal Serum/Plasma

Human serum and plasma contain various classes of RNA molecules which have considerable potential as stable and reliable, minimally invasive, easily accessible biomarkers [3,230,231]. In blood, circulating miRNAs exist in a vesicle-free form associated with ribonucleoprotein complexes [232,233,234,235] or are enclosed in extracellular vesicles [236]. Their concentration and pattern change dynamically in the placenta [50] and blood during the course of pregnancy [237].

In the blood of pregnant women, the RNA of male fetoplacental origin has been identified by detecting a Y chromosome-specific protein-coding RNA [238]. Circulating microRNAs in blood have been implicated in cell-to-cell communication and provide useful biological information about communication between mother, fetus, and placenta [237]. Their expected function is a transmission of signals from the placenta to distant organs and cells, where they might bind to specific surface receptors. The fetal contribution to the RNA pool in maternal plasma is 3.7% in early pregnancy, increasing to 11.28% in late pregnancy [239].

Non-invasive blood tests that provide information about fetal development and pregnancy pathologies have the potential to revolutionize prenatal care. Most of the circulating placenta-derived miRNAs in maternal serum have trophoblast origin and may reflect the status of the placenta. Whitehead et al. [240] discussed the potential of circulating placental RNAs to non-invasively predict pregnancy complications. Therefore, they might serve as novel biomarkers for a wide panel of pregnancy disorders such as preeclampsia, intrauterine growth restriction, and imminent abortion [3]. They can be used to predict gestational age (with comparable accuracy to ultrasound but at substantially lower cost), risk of preterm delivery [241], gynecological diseases, as well as the outcome of in vitro fertilization [242,243] and early [42] or recurrent miscarriage [244].

### Free RNAs and Pathologies

The circulating lncRNAs XLOC_014172 and RP11-230G5.2 serve as a fingerprint for GDM [245] and GDM associated with the risk of macrosomia [246]. Maternal pre-pregnancy overweight, which can lead to different pregnancy complications, is associated with circulating miRNAs in early-mid pregnancy that have also been associated with adipogenesis [247]. Low vitamin B12 levels during pregnancy alter adipose-derived circulating miRNAs, which may mediate an adipogenic and insulin-resistant phenotype, leading to obesity [248]. Maternal fasting seems not to affect circulating placenta-specific transcripts [249]. Gardiner et al. [250] found 55 miRNAs in serum significantly altered through alcohol use during pregnancy. Circulating RNAs can predict infant growth deficits after prenatal alcohol intake [251].

Significantly higher levels of miRNA-200b and miRNA-429 were found in the sera of anovulatory women [252]. In the plasma of patients who later developed PE, 25 small ncRNAs have been identified which are differentially expressed, some of which indicate early-onset PE [253,254] and the severity of the disease [255]. Jung et al. [256] have found circulating RNA involved in the pathogenesis of PE in amniotic fluid. Five significantly differentially regulated circulating lncRNAs have been identified in the plasma of pregnant women with typical fetal congenital heart defects [257]. Moreover, other cell-free RNAs and circulating proteins seem to be potential biomarkers for this disease [258]. A unique circulating placental transcriptome is detectable in maternal blood in pregnancies destined to develop late-onset fetal growth restriction [259]. Carbone et al. [260] have reviewed and discussed the usefulness of circulating RNAs in diagnostics in pregnancy. See also Table 4.

## 7. Extracellular Vesicles

**Figure 3:**
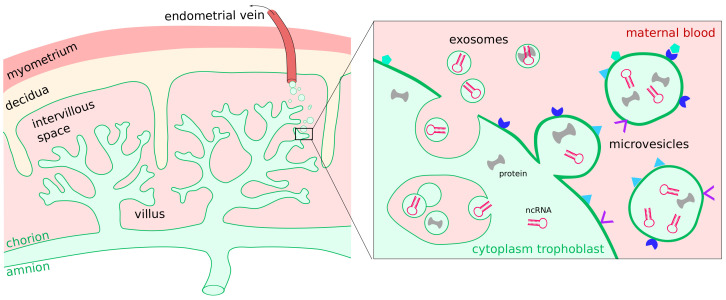
Extracellular vesicles are released from the syncytiotrophoblast cells into the maternal blood. Microvesicles and exosomes can contain proteins, metabolites and non-coding RNAs.

Extracellular vesicles (EVs) are membrane-enclosed packages that ensure the stability of RNA through separation from RNases in the circulation [263]. They are formed by a variety of cell types leading to unique molecular compositions. EVs modulate vascular homeostasis, facilitate immunotolerance, transport regulatory miRNA to target cells, and may stimulate immunity to tumor cells [264,265]. The largest fraction of EVs detectable in the serum of pregnant women is derived from the syncytiotrophoblast (Figure 3) [266,267,268]. It includes exosomes, microvesicles, apoptotic bodies, and syncytial nuclear aggregates for intensive and efficient feto-maternal communication [267,269,270].

Syncytial knots, the classical term for syncytial nuclear aggregates of approximately 2μm diameter (macro EVs) have been detected in the human lung and have been under morphological investigation since the late 19th century [271]. During the last decade, modern methods have allowed more refined analyses of EVs. Microvesicles are 50–1000 nm in size and passively released from the syncytiotrophoblast membrane while exosomes, 30–100 nm, are actively secreted through a fusion of intracellular multivesicular bodies with the plasma membrane. Placenta-derived microvesicles and exosomes are characterized through their membrane expression of placental alkaline phosphatase (PLAP) [272]. This property can be used for quantification [2].

Syncytiotrophoblast EVs circulate through the maternal organism which enables the trophoblast to communicate with cells in distant regions of the body. Their number increases with progressing gestation [267]. Microvesicles and exosomes have different biological functions, cargos and modes of production, reviewed in [273]. The complex cargos consist of bioactive mediators, such as proteins, DNA, mRNA transcripts, miRNAs, other ncRNA, and lipids [270]. Numerous studies have investigated the EV composition (reviewed for example by Chamley et al. [274] and Tong et al. [267]), but the exact mechanisms of interaction with cells are widely unknown. Exosomes secreted by the blastocyst seem to influence the gene expression and receptivity of endometrial cells to prepare and control implantation [275]. Further reviews about EVs during pregnancy can be found [276,277].

### EVs in Pregnancy Pathologies

Syncytiotrophoblast EVs contribute to maternal tolerance towards the fetus, and their dysregulation may lead to pathologies such as PE [19]. Throughout pregnancy, in presymptomatic women who develop PE, the concentration of total exosomes and placenta-derived exosomes in maternal plasma is significantly elevated [261]. In PE, the level of total exosomal miRNA is increased [278] and EVs have a unique miRNA profile [279]. The level of hsa-miR-210 is elevated in women with PE, and even more in the severe form [278]. Exosomal miR-15a-5p promotes the progression of PE [262]. miR-141 is abnormally expressed in PE placentas and elevated levels of miR141 can be transferred from trophoblast to immune cells by release and internalization of EVs [18,280]. However, the influence of EVs on immune cells needs further investigation (reviewed by Redman and Sargent [281] and Delorme-Axford et al. [282]). Their elevated number and altered miRNA content damage endothelial cells, resulting in endothelial dysfunction and disturbed angiogenesis (reviewed in [70,270,283,284]). The uptake of syncytiotrophoblast EVs into primary human coronary artery endothelial cells has been demonstrated, as well as the transfer of placenta-specific miRNAs into their endoplasmic reticulum and mitochondria which causes endothelial damage and oxidative and endoplasmic reticulum stress [285]. Placenta-associated exosomal miR-155 in serum from patients with PE suppresses endothelial nitric oxide synthase expression in endothelial cells [286]. In a human ex vivo placenta perfusion model of PE induced through hemoglobin, syncytiotrophoblast EVs show an altered miRNA content, in particular of the placenta specific mir-517a and mir-517b, members of the chromosome 19 miRNA cluster [287]. Further, the number of exosomes is higher in trophoblast cells cultured under 1% compared to 8% oxygen. Exosomes from trophoblast cells cultured at 8% oxygen increase endothelial cell migration, whilst exosomes cultured at 1% oxygen decrease it [288].

The extrusion of EVs by the placenta is also significantly increased in GDM [289]. Gillet et al. [290] identified 10 miRNAs up-regulated in GDM influencing trophoblast proliferation and differentiation, insulin secretion and regulation, and glucose transport. Nair et al. [291] showed that exosomal circular RNAs can affect skeletal muscle insulin sensitivity. Fallen et al. [292] identified a number of miRNAs in EVs that can be used as biomarkers for preterm labor that may reflect the pathological changes of the placenta. Decidual macrophage-derived miR-153-3p, as a regulator of trophoblast functions, is involved in recurrent spontaneous abortion [293]. Furthermore, infections, such as malaria or HIV, can lead to changes in trophoblast EV composition as recently demonstrated [19]. Trophoblastic exosomes exhibit the highest antiviral activity, containing trophoblastic miRNAs expressed from the chromosome 19 miRNA cluster (C19MC) [282,294]. It has been shown that miRNA transfected into trophoblastic cell lines are expressed at different levels into their microvesicles and exosomes. They can be transferred to T cells and influence their behavior [18]. Rice et al. [295] found that the trafficking of EVs is bi-directional: macrophage- (but not monocyte)-derived EVs induce the release of pro-inflammatory cytokines by the placenta. See also Table 4.

## 8. Conclusions

The human placenta, and especially trophoblast cells, express huge amounts of all classes of ncRNAs. They seem to be involved in the physiological development of the organ, but also in the differentiation and regulation of trophoblasts and other cells. NcRNAs function inside their cells of origin, but also, they seem to be actively and selectively released via different kinds of EVs. These vehicles can be incorporated by different cell types in the placenta or in distant organs where they can influence the behavior of recipient cells. The formation and uptake of EVs can be affected by pregnancy-related pathologies or independent diseases. Thereby, they may be involved in the respective pathomechanism and lead to local and distant symptoms. NcRNAs circulating in the maternal bloodstream have a great potential for non-invasive diagnostics beginning from very early pregnancy potentially leading to the development of novel early treatment strategies.

## Figures and Tables

**Table 1:** **Tools for ncRNA identification.** None of the existing tools for ncRNA identification have been developed specifically for the placenta. Therefore, a generic overview of tools for identification of ncRNAs is given: (**a**) by homology; (**b**) de novo; and (**c**) hybrid approach by including experimental work. Please note: this is a selection of available tools.

Tool	Descripton	Citation
**(a) Homology-based tools for ncRNA identification**		
Blast/RNAcentral	sequence based search	
Infernal/Rfam	prediction based on covariances of secondary structures	
**(b) Tools for de novo identification of ncRNAs**		
RNAz	predicting structurally conserved and thermodynamically stable RNA secondary structures in multiple sequence (genome) alignments	[22]
QRNA	prediction based on comparative genome sequence analysis	[23]
RNAsamba	tool to predict the coding potential of RNA molecules from sequence information using a neural network-based that models both the whole sequence and the ORF to identify patterns that distinguish coding from non-coding transcripts	[24]
FEELnc	alignment-free program that accurately annotates lncRNAs based on a Random Forest model trained with general features such as multi k-mer frequencies and relaxed open reading frames	[25]
LGC	discriminating lncRNAs from protein-coding RNAs across diverse species that range from plants to mammals	[26]
CPAT	novel alignment-free method: recognizes coding and noncoding transcripts from a large pool of candidates	[27]
COME	identification and characterization of novel lncRNAs	[28]
PLEK	algorithm to distinguish lncRNAs from messenger RNAs (mRNAs), in the absence of genomic sequences or annotations	[29]
PhyloCSF	comparative genomics method that analyzes a multispecies nucleotide sequence alignment to determine whether it is likely to represent a conserved protein-coding region, based on a formal statistical comparison of phylogenetic codon models	[30]
**(c) Identification of ncRNAs with the help of transcriptomic data**		
lncRScan-SVM	tool for predicting the lncRNAs (classifying protein coding and lncRNA transcripts using support vector machine)	[31]
slncky	lncRNA discovery tool that produces a high-quality set of lncRNAs from RNA-sequencing data and further uses evolutionary constraint to prioritize lncRNAs that are likely to be functionally important	[32]
CNCI	effective for classifying incomplete transcripts and sense–antisense pairs (highly accurate classification of transcripts assembled from whole-transcriptome sequencing data in a cross-species manner, that demonstrated gene evolutionary divergence between vertebrates, and invertebrates, or between plants, and provided a long non-coding RNA catalog of orangutan)	[33]
CREMA	tool that can be used to rank long non-protein coding RNA predictions for use in conjunction with gene expression studies	[34]

**Table 2:** miRNAs and their role in preeclampsia (selection). For more details see the references and for a larger overview the reviews [91,92,93,94,95].

miRNA	Function	Citation
let-7d	down-regulation inhibits the proliferation and invasion of trophoblast cells	[71]
miR-15b	inhibits trophoblast cell invasion and endothelial cell tube formation by suppressing the expression of argonaute 2	[83]
miR-17, miR-20a, miR-20b	are differentially expressed in PE, regulating EPHB4 and ephrin-B2 expression in trophoblast and endothelial cells via the same “seed” sequence	[98]
mir-20b	may contribute to PE through inhibiting proliferation, invasion and migration of placental trophoblast cells by targeting MCL-1	[99]
miR-22	up-regulation is modulating production of androgen and estrogen and up-regulated in PE placenta	[100]
miR-30a-3p	expression is significantly increased in PE and might be involved in the pathogenesis by targeting IGF-1 and regulating the invasion and apoptosis of trophoblast cells	[78]
miR-34a	hypo-methylation of the miR-34a promoter is associated with PE and PE severity	[101]
	regulates trophoblast invasion through the Notch signal transduction	[102]
	contributes to trophoblast cell apoptosis in PE by targeting BCL-2	[87]
	down-regulation miR-34a-5p improves invasion and migration of trophoblast cells by targetting SMAD4	[103]
miR-93	inhibits MMP-2 and reduces migration and invasion of immortalized trophoblast cells	[76]
miR-128a	induces apoptosis of HTR-8/SVneo cells and thus may contribute to PE	[86]
miR-181a-5p	is increased in both the plasma and placenta of severe PE patients and suppresses the invasion and migration of HTR-8/SVneo cells by directly targeting IGF2BP2	[74]
	up-regulation induces apoptosis, and suppresses invasion in HTR-8/SVneo and JAR cells.	[104]
miR-134	down-regulates ITGB1 and inhibits infiltration of trophoblast cells in placenta of patients with PE	[79]
miR-135a-5p	promotes migration and invasion of trophoblast cells through targeting β-TrCP	[105]
miR-137	reduces the proliferation and migration of trophoblast cells by targeting ERRα	[72]
miR-141	up-regulated in PE and regulates trophoblast (JEG-3 and HTR-8/SVneo) proliferation and invasion and intercellular communication vie EVs	[18]
	hypoxia-induced microRNA-141 regulates trophoblast apoptosis, invasion, and vascularization by blocking CXCL12β/CXCR2/4 signal transduction	[106]
	miR-141-5p regulates ATF2 via effecting MAPK1/ERK2 signaling to promote preeclampsia	[107]
miR-144	may play an important role in the pathogenesis of PE through targeting PTEN in trophoblastic cells	[108]
	MicroRNA-144-3p may participate in the pathogenesis of preeclampsia by targeting Cox-2	[109]
miR-195	could promote cell invasion via directly targeting ActRIIB in trophoblast cells	[82]
	is suggested to regulate PE by affecting placental proliferation, apoptosis, and angiogenesis	[110]
miR-200	miR-200c,-20a and -20b are involved in hydrogen sulfide stimulation of VEGF in trophoblasts	[111]
miR-203	significantly increased in PE placenta inhibiting vascular endothelial growth factor A (VEGFA)	[112]
miR-218	contributes to PE by targeting LASP1 to inhibit trophoblast invasion	[81]
miR-299	up-regulation suppresses the invasion and migration of HTR-8/SVneo trophoblast cells partly via targeting HDAC2	[75]
miR-320a	overexpression in PE placenta inhibits trophoblast cell invasion by targeting estrogen-related receptor-gamma (ERRγ), but not migration or proliferation	[80]
	upregulation inhibits proliferation and invasion of trophoblast cells by targeting IL-4	[113]
miR-454	promotes the proliferation and invasion of trophoblast cells by inhibiting EPHB4 expression	[73]
	promotes the proliferation and invasion of trophoblast cells by downregulation of ALK7	[114]
miR-520c-3p	is suppressing inflammasome activation and inflammatory cascade by down-regulating NLRP3	[115]
miR-520g	is suppressing the migration and invasion of trophoblast via at least partial inhibition of MMP2 translation inhibition	[77]
miR-4421	is highly expressed in PE, which may promote the progression of PE by down-regulating the expression of CYP11B2	[116]
miR-125b	involved in early PE development through regulation of Trop-2 expression	[117]

**Table 3:** Long non-coding RNAs involved in pathologies. NN—no name.

LncRNA	Function	Citation
Malat-1	down-regulated in preeclampsia, regulates proliferation, apoptosis, migration and invasion of JEG-3 choriocarcinoma cells	[173,174]
	affects the migration and invasion of trophoblast cell by regulating FOS expression	[175]
	regulates trophoblast cells migration and invasion via miR-206/IGF-1 axis	[176]
LOC391533, LOC284100, CEACAMP8	dysregulation seems to be associated with preeclampsia	[15]
linc00473	down-regulated in the placenta of patients with severe PE. knockdown in trophoblast cell lines significantly inhibites cell proliferation and promotes apoptosis, whereas overexpression stimulates trophoblast proliferation. Further, linc00473 inhibited the expression of tissue factor pathway inhibitor 2 (TFPI2) through binding to lysine-specific demethylase 1 (LSD1)	[177]
	mediates decidualization of human endometrial stromal cells in response to cAMP signaling	[178]
	downregulation facilitates trophoblast cell migration and invasion via the miR-15a-5p/LITAF axis in pre-eclampsia,	[179]
	Linc00473 mediates regulation of Wnt/β-catenin signaling pathway by miR-424-5p and affects invasion and migration of the trophoblastic cell line HTR-8/SVneo	[180]
PRNCR1	promotes the progression of PE by modulating the MAPK signaling pathway	[181]
CCAT1	is highly expressed in PE and can promote the progression of PE by inhibiting the expression of CDK4	[182]
MEG3	is lower expressed in the placenta of patients with PE, and its regulation of trophoblast cell epithelial-mesenchymal transition via the TGF-β pathway inhibitor SMAD7 may be the molecular mechanism involved in the pathogenesis of PE	[183]
TUG1	is modulating proliferation in trophoblast cells via epigenetic suppression of RND3	[184]
	is modulating proliferation, apoptosis, invasion, and angiogenesis via targeting miR-29b in trophoblast cells	[185]
	is regulating the migration and invasion of trophoblast-like cells through sponging miR-204-5p	[186]
lnc-DCs	overexpression in dendritic cells mediates their maturation by phosphorylating STAT3 and induces the over-maturation of decidual dendritic cells in PE and leads to an increase in Th1 cells	[187]
RPAIN	regulates the invasion and apoptosis of trophoblast cell lines via complement protein C1q	[188]
ATB	down-regulated in PE placentas which was found to decrease migration, proliferation, and tube-formation of HTR-8/SVneo cells	[189]
	functions as a competitive endogenous RNA of miR-651-3p to regulate YY1 on progress of spiral artery remodelling	[190]
uc.187	is up-regulated in preeclampsia and modulates proliferation, apoptosis, and invasion of HTR-8/SVneo cells	[191]
SPRY4-IT1	modulates trophoblast cell invasion and migration by affecting the epithelial-mesenchymal transition	[192]
	up-regulation modulates proliferation, migration, apoptosis, and network formation in HTR-8SV/neo cells	[16]
NN	An lncRNA within intron 3 of the *STOX2 gene* which seems to regulate an essential trophoblast differentiation pathway	[193]
XIST	has a role in X chromosome inactivation in females, a process that is paternal specific in the trophoblast and random in the fetus	[194,195]
lncRHOXF1	is the first example of an lncRNA from the X chromosome that regulates the host response to viral infections in human placental progenitor cells	[196]
LncRNA-TCL6	plays a role in early abortion by inhibiting placental implantation via the EGFR pathway	[197]
LncRNaIGF2-AS	plays a role in recurrent miscarriage by regulating trophoblast functions	[198]
PVT1	is down-regulated in GDM and PE	[199]
HOTAIR	plays an important role in suppressing angiogenesis of the human placenta by inhibiting the expression of VEGFA	[171]
NEAT1	is increased in intrauterine growth retardation (IUGR) placentas but the pathomechanism is not yet clear; up-regulation is inducing apoptosis in HTR-8/SVneo cells	[200,201]

**Table 4:** Role of free circulating and exosomal ncRNAs in pregnancy-related diseases (selection).

Disease	Circulating ncRNA	Citation
Early pregnancy loss	up-regulated hsa-let- 7c, hsa-miR-122 and down-regulated hsa-miR- 135a in plasma	[42]
Recurrent miscarriage	miR-27a-3p, miR-29a-3p, miR-100-5p and miR-127-3p are increased and miR-486-5p decreased in plasma	[244]
Fetal growth restriction	up-regulated miR-16-5p, miR-103-3p, miR-107-3p, and miR-27b-3p in plasma	[141]
Fetal congenital heart defects	lncRNAs ENST00000436681, ENST00000422826 are up-regulated and AA584040, AA709223 and BX478947 down-regulated in plasma	[257]
Placenta accreta spectrum	down-regulated miR-139-3p, miR-196a-5p, miR-518a-3p, and miR-671-3p in serum	[147]
Gestational diabetes mellitus	miR-223 and miR-23a are up-regulated in plasma	[245]
Preeclampsia	hsa-circ-0036877 is up-regulated in blood	[223]
Preeclampsia	circCRAMP1L circulating levels are significantly lower in plasma	[224]
Preeclampsia	miR-215, miR-155, miR-650, miR-210, miR-21 are up-regulated, and miR-18a, miR-19b1 down-regulated in plasma	[255]
Preeclampsia	hsa-miR-486-1-5p and hsa-miR-486-2-5p are up-regulated in exosomes	[261]
Preeclampsia	hsa-miR-210 is up-regulated in exosomes	[258]
Preeclampsia	miR-15a-5p is up-regulated in exosomes	[262]
